# The global health concept of the German government: strengths, weaknesses, and opportunities

**DOI:** 10.3402/gha.v7.23445

**Published:** 2014-02-13

**Authors:** Kayvan Bozorgmehr, Walter Bruchhausen, Wolfgang Hein, Michael Knipper, Rolf Korte, Oliver Razum, Peter Tinnemann

**Affiliations:** 1 Department of General Practice & Health Services Research, University of Heidelberg, Heidelberg, Germany; 2 Institute of History, Theory and Ethics in Medicine, Aachen University, Aachen, Germany; 3 Institute of Latin American Studies, German Institute of Global and Area Studies (GIGA), Hamburg, Germany; 4 Faculty of Medicine, Institute of the History of Medicine, University Giessen, Giessen, Germany; 5 Faculty of Medicine, Institute of Hygiene and Environmental Health, University Giessen, Giessen, Germany; 6 Department of Epidemiology & International Public Health, School of Public Health, Bielefeld University, Bielefeld, Germany; 7 Department of International Health Sciences, Institute for Social Medicine, Epidemiology and Health Economics, Charité-University Medical Centre Berlin, Berlin, Germany

**Keywords:** global health, foreign policy, health policy, governance, globalisation, policy analysis

## Abstract

Recognising global health as a rapidly emerging policy field, the German federal government recently released a national concept note for global health politics (July 10, 2013). As the German government could have a significant impact on health globally by making a coherent, evidence-informed, and long-term commitment in this field, we offer an initial appraisal of the strengths, weaknesses, and opportunities for development recognised in this document. We conclude that the national concept is an important first step towards the implementation of a coherent global health policy. However, important gaps were identified in the areas of intellectual property rights and access to medicines. In addition, global health determinants such as trade, economic crises, and liberalisation as well as European Union issues such as the health of migrants, refugees, and asylum seekers are not adequately addressed. Furthermore, little information is provided about the establishment of instruments to ensure an effective inter-ministerial cooperation. Finally, because implementation aspects for the national concept are critical for the success of this initiative, we call upon the newly elected 2013 German government to formulate a global health strategy, which includes a concrete plan of action, a time scale, and measurable goals.

To date, German governmental institutions have paid little attention to the concept of global health, which is an emerging policy field (1). The country's involvement in the field (2) has been referred to as literally invisible (3) and in a stage of infancy (4). For this reason, the authors welcome the launch of a first national concept document for global health politics entitled *Globale Gesundheitspolitik gestalten – Gemeinsam handeln – Verantwortung wahrnehmen* (Shaping Global Health – Taking Joint Action – Embracing Responsibility – 10 July 2013) (5).

In line with other countries that have already launched national global health strategies – such as Switzerland (2006), the United Kingdom (2008), Norway and Japan (2010), Sweden (2011), as well as the European Union (6) (EU) – with the release of this document, the German federal government also expresses its commitment to advancing health and wellbeing on a global scale.

The primary goal stated in the government's national concept (5) is to make an active and consolidated contribution to solving pressing global health challenges of our time. It defines five key areas of action where Germany can play a vital role in improving health on a global level: 1) tackling cross-border threats to health; 2) strengthening health systems worldwide (by enhancing systems of social health protection and improving public access to health care services); 3) ensuring intersectoral cooperation for health; 4) promoting/strengthening health research and the health care industry; and 5) strengthening the global health architecture (5).

We maintain that as an important voice in the international community, Germany has a special responsibility towards global health both at the European and the global level. The government has traditionally embraced its responsibility for health in developing countries primarily via bilateral (and to a lesser extent multilateral) aid. Health policy at the European level, in contrast, has been mainly embraced via legal frameworks within the EU. Presenting a coherent, evidence-informed, and far-sighted global health concept that overcomes these North–South binaries and draws upon the strengths of other countries’ recent strategies could thus have a significant impact on health globally.

The effort of the German government to prepare the presented global health concept is highly valued and its release has already initiated debate about gaps and ambitions (4, 7). In order to provide a rationale for proposals for further improvement related to the concept, the strengths, weaknesses, opportunities, and threats identified in the national concept have been analysed and are detailed in this paper. Four authors (KB, WB, MK, OR) independently read the concept of the federal government with the task to evaluate the major strengths and weaknesses in the document. Common issues identified by more than one author were fed into a preliminary list of items considered to be most important. This list was reviewed and scrutinised until all authors reached consensus.

We identified three major strengths relating to important issues on the global health agenda: Firstly, a clear and unequivocal commitment to Universal Health Coverage (UHC) (8) based on the ‘human right to health’ (HR2H) (9) approach, including health systems strengthening; equality and equity in access to quality health care; protection against catastrophic health expenditure (10); and the acknowledgement of the regulatory role of states in this context.

Secondly, there was an equally clear and unequivocal commitment to strengthen the leadership role of WHO as the sole coordinating agency for global health policy. This includes, in line with the Paris Declaration on Aid Effectiveness, a clear commitment to counter attempts to create new organisations and initiatives in the (global) health sector duplicating existing mandates and tasks. Noteworthy is particularly the commitment to strengthen the ‘core mandate’ of the WHO in setting *binding* norms and standards for its member countries *and* all other actors in global health – an issue widely discussed in the context of a Framework Convention on Global Health (11).

Thirdly, the national concept aims to strengthen intersectoral cooperation (12) in order to improve population health by adopting a public health approach instead of an individual, exclusively biomedical approach.

On the contrary, the national concept contains some important gaps and weaknesses. For example, no reference is made to the important debates on the impact of intellectual property rights on access to medicines and innovation in health. In particular, policy coherence with regard to the WHO General Strategy and Plan of Action on Public Health, Innovation and Intellectual Property (GSPoA) and its follow-up, which are central for the problem of adequate incentives for medical research, remains unaddressed (13). In this particular context, the government's concept falls short of the EU council conclusions (6). With the elaborations on falsified medicinal products, ignoring the role of generics and compulsory licenses, the government's concept (consciously or unconsciously) adopts lines of arguments of private pharmaceutical industries (13). A progressive IPR policy, coherent with international resolutions (14), would resolve that 1) no trade or investment treaty initiates intellectual property rights that go beyond those articulated under the multilateral TRIPS agreement, and that 2) the specific wording of the 2001 Doha Declaration on the right to issue compulsory licences be written into all future trade and investment treaties.

Given that strengthening the German health care industry is an explicit primary goal of the concept (5, p. 34), the above discrepancy with international policy recommendations (6, 13, 14) might not be surprising. The national concept places a particular emphasis on the promotion of the ‘Export Initiative [for the German] Health Industry’ and the ‘German Healthcare Partnership’ (p. 36). Given that poor health in low-income countries is a problem mostly driven by inequity and social determinants (12) rather than by a lack of technology, there is a risk that the aim of utilising ‘the strengths of the German health care industry for the benefit of global health’ (5, p. 34) diverts scarce resources in low- and middle-income countries to costly technologies from urgently needed social interventions promoting equity.

Significantly, the national concept provides an extensive inventory of past and on-going conventional approaches to international health (15), reflecting a ‘sending culture’ of resources, competencies and experts to ‘developing’ countries with an over-emphasis on bilateral agreements (4). This lens tends to neglect the rise and importance of truly ‘global’ (16) issues such as economic crises (17), international trade (18) and liberalisation (19), as well as the political economy of health (20), including global inequity (12). Addressing the health impacts of these global determinants (16) should be considered a primary motive or ‘leading thought’ of any global health concept.

A comprehensive, systemic approach – which acknowledges that global health starts ‘at home’ – would move towards coherence with ratified UN resolutions on UHC (21) and HR2H (22) and address the serious limitations related to the right to the highest attainable state of health for migrants, refugees and asylum seekers in the EU, including Germany (23). It would also outline a far-sighted strategy to stimulate global health research and education in Germany beyond isolated programs.

Importantly, the government's commitment to strengthen WHO (5, pp. 38–39) details out several measures to improve the organisation's efficiency (by improving budget setting-procedures, goal-orientation and financial management, transparency, internal control mechanisms, and implementation of regular external evaluation measures) but remains vague as far as other important organisational aspects are concerned. While efficiency is important, the organisation's effectiveness depends, not least, on financial independence as far as goal and priority setting is concerned. Thus, any serious commitment to strengthening WHO should – in line with the EU council's conclusions (6) – declare a willingness to increase non-earmarked financial contributions in support of the institution. Attempts of internal structural reform should be based on solid evidence that this is an adequate strategy to strengthen the institution's capacity of effectively fulfilling its mandate in contemporary complex-adaptive systems.

Finally, the concept of the federal government would greatly benefit from a transparent, operational and binding strategy on how to organise the all-important inter-ministerial cooperation (1) in the national context, particularly between the Ministries of Health (BMG), Development and Economic Cooperation (BMZ), Foreign Affairs (AA), Finances (BMF), Economy (BMWi), Justice (BMJ), and Research and Education (BMBF). Within a commitment to ‘achieve the greatest possible degree of consistency among the policymakers responsible for questions related to global health’ (5, p. 41), the federal government explicitly refers to foreign and development policies only, but not to economic policies. A clear strategy is needed on how to interweave global health within interrelated national German policies.

The Swiss ‘Gesundheitsaußenpolitik’ (Health Foreign Policy) already provides several instruments designed for this task: the establishment of a coordinating office for health foreign policy, implementation of bi-annual meetings of inter-ministerial working groups, an annual inter-ministerial conference on health foreign policy, establishment of a coordinating office for global health policy, and the creation of an interdepartmental information platform for global health (24), p. 16). Without institutional innovations the laudable commitment to UHC and HR2H might remain mere rhetoric, since major powerful determinants of health (17) are outside the scope of development politics or health politics.

## Conclusions

The national concept of the German federal government is an important first step towards a coherent national global health policy. Based on our appraisal, we are concerned that the current strategy might fail to achieve its overarching goal of making a consolidated contribution to solving the pressing global health challenges of our time because of the described gaps and weaknesses related to conceptual and implementation issues. We urge the new German government to develop a concrete plan of action to support global health, including a time scale and measurable goals.
